# Simulating Society Transitions: Standstill, Collapse and Growth in an Evolving Network Model

**DOI:** 10.1371/journal.pone.0075433

**Published:** 2013-09-25

**Authors:** Guanghua Xu, Junjie Yang, Guoqing Li

**Affiliations:** 1 State Key Laboratory of Vegetation and Environmental Change, Institute of Botany, Chinese Academy of Sciences, Beijing, China; 2 University of Chinese Academy of Sciences, Beijing, China; 3 State Key Laboratory of Soil Erosion and Dryland Farming, Institute of Soil and Water Conservation, Northwest A&F University, Yangling, Shaanxi, China; Cinvestav-Merida, Mexico

## Abstract

We developed a model society composed of various occupations that interact with each other and the environment, with the capability of simulating three widely recognized societal transition patterns: standstill, collapse and growth, which are important compositions of society evolving dynamics. Each occupation is equipped with a number of inhabitants that may randomly flow to other occupations, during which process new occupations may be created and then interact with existing ones. Total population of society is associated with productivity, which is determined by the structure and volume of the society. We ran the model under scenarios such as parasitism, environment fluctuation and invasion, which correspond to different driving forces of societal transition, and obtained reasonable simulation results. This work adds to our understanding of societal evolving dynamics as well as provides theoretical clues to sustainable development.

## Introduction

The evolving dynamics of society is well worthy of our concern for it signifies whether we would have a sustainable future or a doomed crash. Many of us nowadays may suppose an ever ascending trend of civilization, because modern society is much more complex and has a much larger population compared to ancient ones. However, this could be an illusion. Real societies could take various evolving tracks. For example, there were countless declined or collapsed societies in history. In *Rossiia i Europa* [1], the Russian philosopher Nikolai Danilewski wrote that each civilization has a life cycle, like a perennial plant that has a continued growing period, but would finally decay. Similar theories were proposed by Spengler and Toynbee. More recently, Turchin and his colleagues coined the term “Secular Cycles” to imply the oscillations between population growth and instability in historical societies [2], [3]. Aside from ascending and cyclic patterns, there are also societies that persisted for a long time with only tiny changes in its structure and population. Still, merging of adjacent societies into a bigger one is possible when communication and transportation technologies are sufficiently advanced. All these possibilities made the evolving dynamics of human civilization a great mist.

Various theories that explain the mechanisms under societal dynamics have been developed. Tainter [4] proposed that the complication process that improves a society’s reaction to challenges has a diminishing marginal return, which finally leads to collapse. Diamond [5] proposed that societies have an internal tendency to overshoot ecological capacity. Environmental impact [6] and enemy invasion are also widely known exogenous factors that have great impact on the course of a society. Recently, complex system theories have also been applied to describe societal dynamics [7], among which are the Self-Organized Criticality theory (SOC) [8], [9], the Dual Phase Evolution theory (DPE) [10], [11], the Adaptive Cycle and Panarchy theory [12]–[16].

Such theories have greatly advanced our understanding of societal dynamics. However, they are generally narrative and thus vague. Simulation models can help make ideas more explicit, and different modeling approaches are available. Abundant models are on dynastic cycles, which boast of dynamic details, *e.g.*, interaction between social classes such as farmers, bandits and rulers [17]–[19]; between sowing area, population and number of peasants and handicraftsmen [20]; or between population density, warfare intensity and state resource reserve [21]. These models have successfully demonstrated the sociodemographic cycles for complex agrarian systems. They are, however, too specific, failing to modeling other societal dynamic patterns. A more abstract modeling approach derives from other disciplines such as artificial chemistry, utilizing an evolving network method [22]. Jain and Krishna [23], [24] developed a model system where species populations co-evolve with their network of interaction, crashes and recoveries that arise dynamically can be observed. Models with similar mechanisms have been developed to explain evolution process and Schumpeterian economic dynamics [25]–[27]. The advantage of this type of models is their capability of illustrating the structure changing process, which is the evolving nature of society, thus could be applied to more areas of societal dynamics.

Most of these theories and models capture only one aspect of the complex society evolving dynamics which can have various possibilities. Thus we need a comprehensive approach to understand the essentials of this process, which is to categorize societal dynamics into different patterns and then explore their possibilities within one model. The more patterns this model can demonstrate, the more likely that it has captured the essentials of society evolving dynamics [28], [29]. In the present paper, we develop a model capable of simulating three widely recognized societal transition patterns, *i.e.*, (1) standstill, which means a society maintains its structure and productivity for a considerably long period; (2) collapse, which means sudden decomposition of structure and drop of productivity to an insignificant level; and (3) growth, which means increase in structure complexity and productivity of the society. These patterns are important compositions of the overall societal evolving process, and each can have different causes, for which corresponding scenarios are set in the model. Here society structure and productivity are variables that we believe to be essential of a society. By structure, we mean the component occupations of the society and relationships between them; while by productivity, we mean the total yield of the society, which is proportional to the total population.

### Model Description

Imagine a virtual society (*S*), which is composed of *n* occupations, dwells in a specific environment (*E*). For example, a traditional agricultural society may have grain cultivators, black smithies, bakers, bricklayers, *etc.* Each occupation has a number of engaged inhabitants who must earn their living through the function of the occupation. We suppose the number of inhabitants an occupation feeds is proportional to its productivity. There exists two types of interactions, the first is the interaction between occupations of the society, which we define as unidirectional supporting, *i.e.,* one occupation may promote the efficiency of occupations that it supports, while itself be promoted by some other occupations. Self-supporting is also allowed. These supporting relationships are based on the characteristics of the environment where the society dwells, which could be through market or beyond, and shaped by technology development of the society. The assumption that these interactions are non-negative has its reason. Newly generated occupation harming existing ones in the society would get opposed, and thus very likely be eliminated before it could become a stable component of the society. The temporal scale of the model is coarse enough to allow the omission of such transient occupations. This could be compared to biological evolution. Whereas most mutation characteristics are harmful, they are generally eliminated with failed individuals, while only those neutral and beneficial mutation characteristics are kept. We will refer the occupations and their relations with each other as the structure of the society, and assume it possesses adequate stability, still undergoing impacts from inside and outside of the society from time to time. Furthermore, we suppose transportation and communication technologies of the society accommodate to the scope and characteristic of the environment and no delay happens in these interactions.

The second type of interactions happens between occupations and the environment. To produce effectively, each occupation has a lot of work to do, *e.g.*, exploiting resources from the environment, altering natural processes to assist production, preventing production activities from negative disturbance, and so on. Here we abstract all these activities as fighting against the environment to gain productivity, while the result is determined by the relative competing strength of the occupation against the environment.

In this concept model, we’ve got entities of three levels, *i.e.*, society, occupation, and inhabitant, which constitute a nested hierarchy. As we aim to model long term transitions of society, and the reproduction of inhabitants is comparatively a very fast process, we thus suppose the number of inhabitants of each occupation be determined by the productivity of the occupation, and the total population by the total productivity of the society. As for occupations in the society, we would propose that a society keeps the memory of its occupations, thus destroyed occupations could be regenerated provided the society is in function and other necessary conditions are met. In the following, we will specify and formalize this concept model.

Let *x_i_* be a non-negative real number that represents the productivity of the *i^th^* occupation (which could also be interpreted as the amount of inhabitants it feeds); let *Cs_i_* denote competing strength of occupation *i*, and *Ce_i_* denote restriction strength of environment for occupation *i*, and define change rate of *x_i_* as:
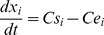
(1)


That is, the productivity of an occupation would increase when its competing strength outweighs the environmental restriction strength, and vice versa. The competing strength of an occupation comes from the supporting of other occupations in the society (and itself, if self-supportable), which marks the intersection of aforementioned two types of interactions. If occupation *j* supports occupation *i*, then occupation *i* gains a fraction of its competition strength *Cs_ij_* from occupation *j*, which we define as:
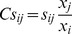
(2)


*s_ij_* is the supporting coefficient of occupation *j* to occupation *i*. Furthermore, we suppose occupations are mutually noninhibitory, and each occupation is not affected by supporting other occupations. Thus, the overall competing strength of occupation *i* (denoted by *Cs_i_*) is given by:
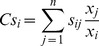
(3)


This means those occupations with more supporting occupations would be more competent, while those without supporting would fail to sustain themselves. Some occupations have to rely on others (*e.g.*, bricklayers cannot feed themselves); some can mainly support themselves (*e.g.*, grain cultivators) but still get support from other occupations (*e.g.*, tools made by black smith help grain cultivators a lot). To favor our following analysis, we add a constraint to the structure of society, *i.e.*, from each occupation in the society, there exists supporting to each other occupation, directly or mediated by other occupations.

The restriction from the environment to an occupation is affected by the characteristics of the environment, the total population of the society, and the features of the occupation. As the total population of the society increases, it would become more difficult to get needed resources or perform other production related activities well, which means increasing restriction from the environment to the occupation:
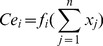
(4)


Here *f_i_* is an incremental function.

Combining Equation 1–4, we get the final equation that governs the dynamics of an abstract society S with *n* occupations:
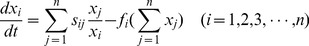
(5)


Generally, occupations in the model society are mutually benefiting, *i.e.*, helping each other with their competition against the environment to earn living. Nevertheless, indirect competition mediated by the environment also exists, *i.e.*, as total population of the society grows, each occupation would bear more pressure from the environment.

There are two major differences between this system and those replicator equations used in chemical system modeling [24], [30]: first, *X_i_* is inverse proportional to its growth rate, which gives prominence to the effect of supporting; second, the second term of *r.h.s* is not dilution flux, thus our system is capable of model society expansion.

Before specifying parameters and running simulations, some simplifications to Equation 5 are necessary. First, let the coefficient of supporting be equal, *i.e.*:
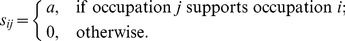
(6)


Here *a* denotes the supporting coefficient.

**Figure 1:**
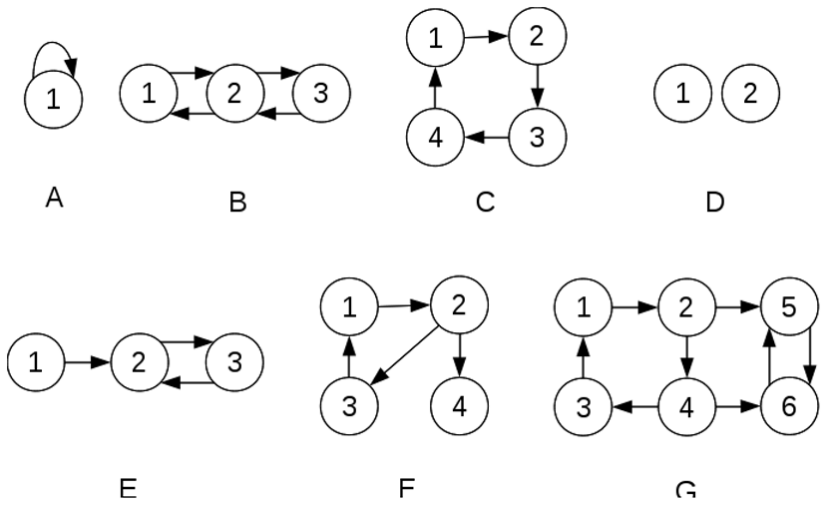
Structure of virtual society, which could be represented by a graph composed of all the occupations of the society as nodes and interactions between them as edges. By our definition, each occupation in the society is supported (directly or mediated by other occupations) by each other occupation in the society, *i.e.*, the graph should be irreducible to denote the structure of a legal society (this does not applies to derived society). Solid arrows in the figure denote supporting relationships. A, B, C are irreducible graphs, and thus represent legal society, while D, E, F, G are not. For D, the two occupations are not connected; for E, occupation 1 is not supported; for F, occupation 4 doesn't support other occupations; for G, occupation 5 and 6 do not support other occupations.

Second, a random 0–1 matrix C is used to represent supporting relationships. Its element *c_ij_* is set to 1 (*i.e.*, occupation *j* supports occupation *i* ) with a probability *p_c_*, and 0 otherwise. Furthermore, based on aforementioned definition of society structure (*i.e.*, there exists direct or indirect supporting from each occupation to each other occupation), C is irreducible (Figure 1).

Third, all occupations in the society share the same environmental restriction function, which is:
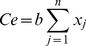
(7)


Here *b* is the environmental restriction coefficient.

After these steps, Equation 5 is converted to the following form:
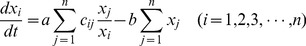
(8)


This is a non-linear differential equation, but with linear character. The equilibrium solutions are the eigenvectors of the matrix C, with their norm restricted by:
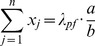
(9)


Because C is irreducible, the Perron-Frobenius theorem guarantees the existence and uniqueness of a real positive eigenvector, which associates with eigenvalue (*λ_pf_*) that equals the spectral radius of the matrix [31]. The minimum real positive eigenvalue C can have is 1 because it is a 0–1 matrix, which means *λ_pf_* ≥1 is a prerequisite for the society to survive in the environment.

Now we introduce dynamic factors into the model. First, an occupation must equip with it a minimum number of practitioners (society inhabitants) to function. If for some reason this is not satisfied, its productivity would drop to 0, and the inhabitants it once feeds would starve to death. Again, for simplicity, we suppose this value be equal for all the occupations in the society, which would be denoted by *limina*. Second, inhabitants could flow between different occupations (for example, some people may get tedious of doing the same job and want to try something new), which we would also term as mutation. We suppose this transformation happens with probability *p_m_* for each occupation, and the transferred portion is equal to *limina,* regardless of the exact population of the occupation. However, an occupation must have a population greater than twice of *limina* to be able to have its crew transformed, otherwise the transformation would destroy the original occupation. We also assume the target occupation of each transformation be actually supported by existing occupations. This is not a problem if the society is in its full occupation state, but there might be occasions when several occupations of the society are not functioning. We suppose the inhabitants carry their share of productivity with them, thus the transformation of inhabitants also means transfer of productivity. Equation 10 describes this transformation.
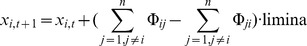
(10)


Φ_ji_ is a random item, which means transfer of productivity from occupation *i* to occupation *j*.

The behavior of the system alternates between developing phase (described by Equation 8) and mutating phase (described by Equation 10). During developing phase, the system is allowed to run enough time to reach its attractor; during mutating phase, random transformation is set on, altering the structure of the system and thus disequilibrating it. The following developing phase, however, would bring it back to equilibrium again, thus on and on. Furthermore, we suppose transformation happens asynchronously and the transformation sequence of the occupations is random.

Now, a virtual society with well defined dynamic characteristics is ready. Before going further, a preliminary demonstration that provides a taste of the model would be beneficial. Suppose a society *S* composed of *n* potential occupations, with their supporting relations set by matrix C, that is randomly generated given the parameter *p_c_*, which denotes the probability of supporting between each pair of occupations. Additional parameters include the supporting coefficient (*a*), environmental restriction coefficient (*b*),and transformation probability of each occupation (*p_m_*) (Table 1). Equation 9 shows that total productivity of a society is determined by supporting coefficient *a*, environmental restriction coefficient *b* and *λ_pf_* of the supporting matrix C. Besides, as our major concern is not the time needed to reach equilibrium, but the general dynamic patterns, we only care about the ratio of *a* and *b* (*i.e.*, *a/b*), not their exact values. When we fix the value of *a/b*, the productivity is proportional to *λ_pf_*, which is determined by *n* and *p_c_*. The bigger these two parameters (thus the more complex the society gets), the higher the steady state productivity would be. We set the parameters of the model according to the following strategy. First, *limina* is set to an arbitrary constant, which is 1.0. Then, we establish the structure of the society, *i.e.*, set the values of *n* and *p_c_*. Third, we set *a/b* properly so that the average productivity of the occupations is in a reasonable interval, which should be higher than *limina*, but not too high. At last, we set *p_m_*. As it is not feasible to get real society data for parameterization, we run the model with different combinations of parameter values. Furthermore, because there are random terms in equation 10, we run each combination with 50 replications to confirm its robustness.

**Table 1:** Model parameters.

Parameter	Description
*a/b*	ratio of supporting coefficient and environmental restriction coefficient
*n_x_*	total occupations in society *x*; options of *x* are *o*, *d*, and *i*, which mean original, derived and invader separately
*p_c_xy_*	probability of an occupation in society *x* supporting an occupation in society *y,* options of *x* and *y* are *o*, *d*, and *i*, which mean original, derived and invader separately
*p_m_*	mutate rate of an occupation
*r_d_*	severity of disturbance

**Figure 2:**
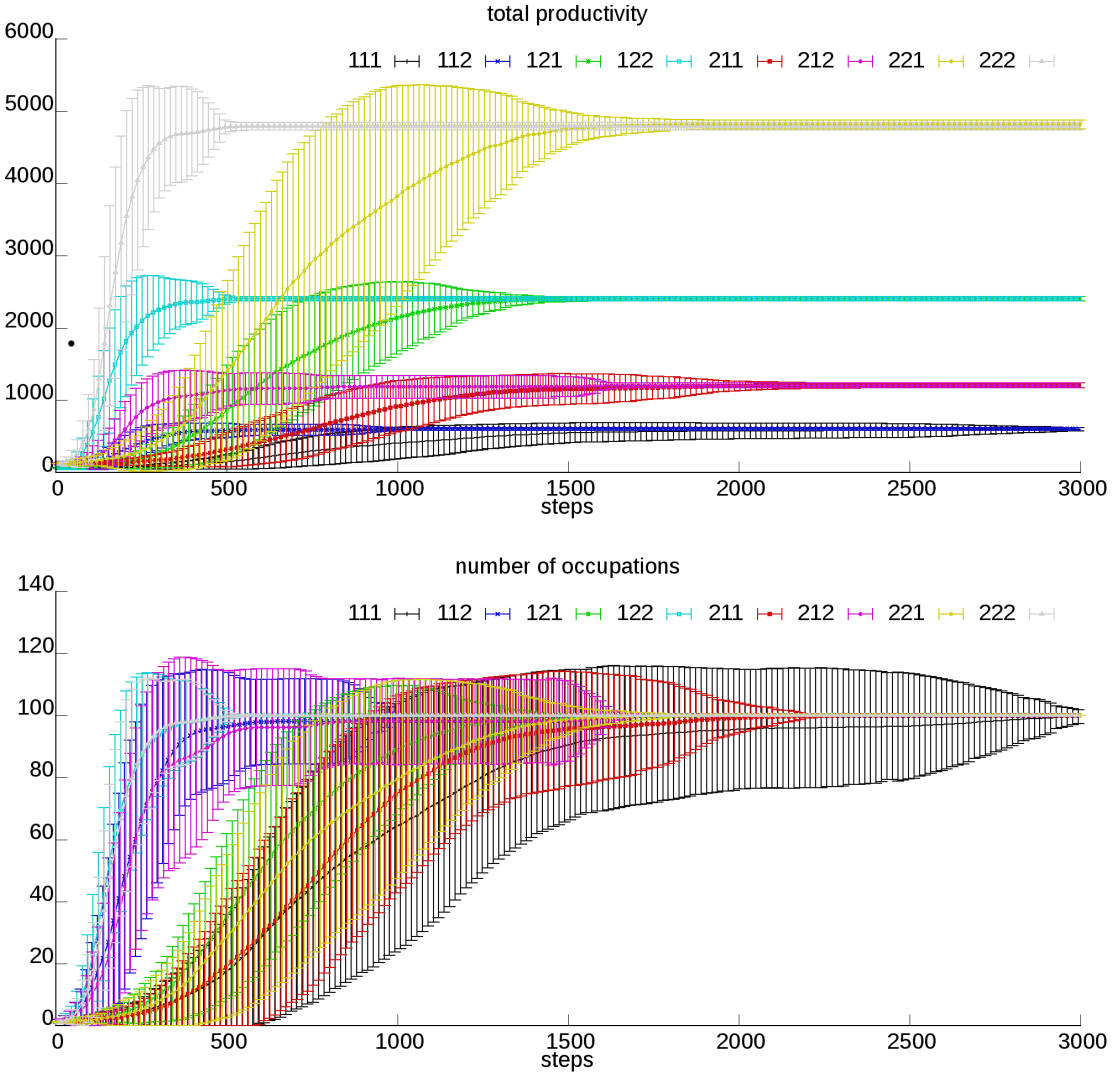
Evolving dynamics of a model society. We set *n* to 100, while *a/b*, *p_c_*, and *p_m_*, are assigned two optional values each, which are: *a/b*1 = 60, *a/b*2 = 120; *p_c_*1 = 0.1, *p_c_*2 = 0.4; *p_m_*1 = 0.01, *p_m_*2 = 0.04. All of the 8 combinations of these parameter values are used for the simulation, and each combination is run with 50 replications. The mean and standard deviation of total productivity and number of occupations of the society are shown. Each combination is termed with four numbers in the figure label, (*e.g.*, 111 refers to the parameter combination “*a/b*1, *p_c_*1, *p_m_*1”, while 221 refers to the combination “*a/b*2, *p_c_*2, *p_m_*1”, *etc*). The 4 final productivity values revealed in the figure correspond to the 4 different combinations of *a/b* and *p_c_*, while for each of these combinations, the larger *p_m_* value corresponds with the short transition time.

**Figure 3:**
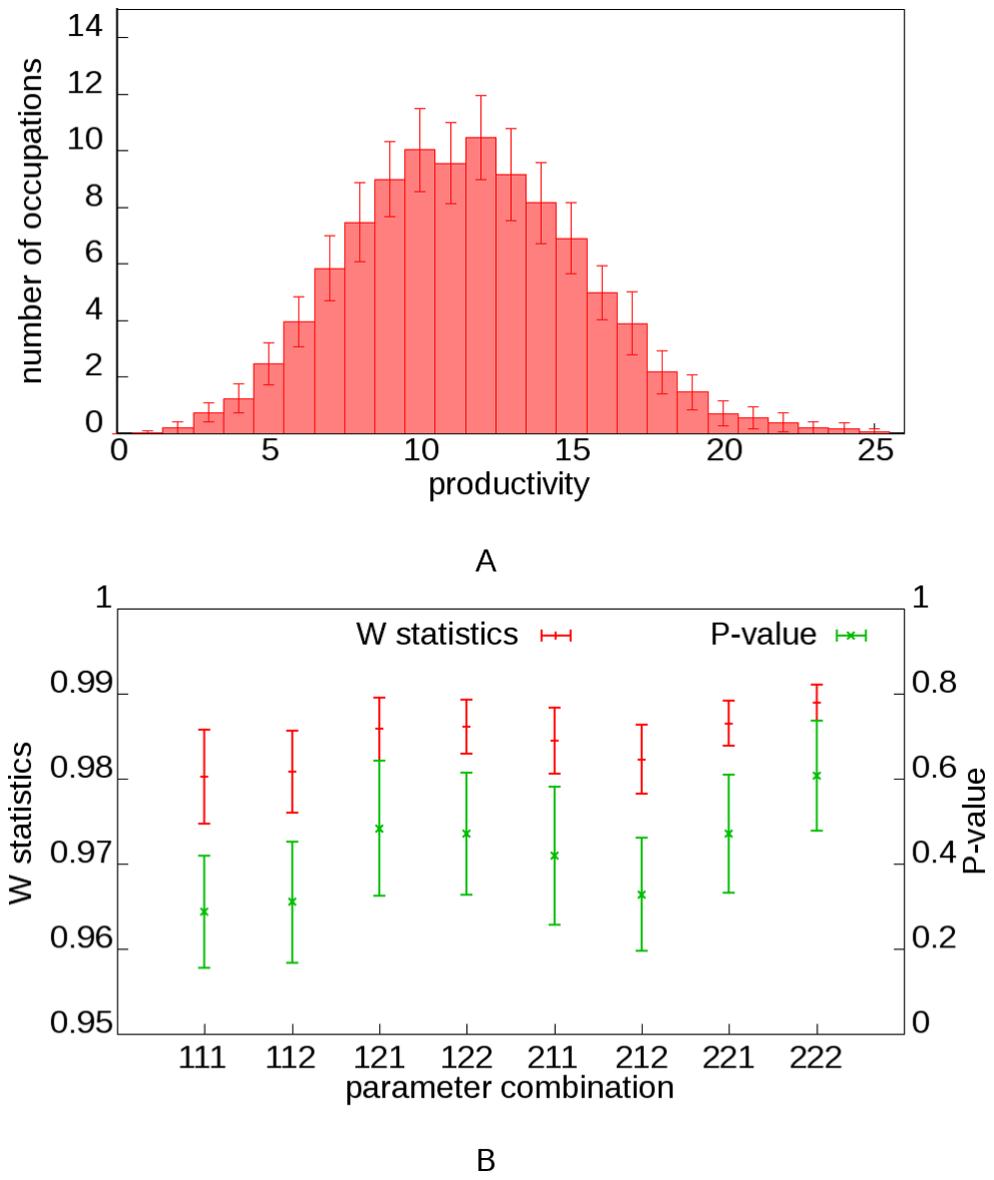
Distribution of steady state productivity. (A) Productivity distribution of the parameter combination 211 as an example. Mean value is used for plotting while standard deviation is represented with error bars. (B) Result of Shapiro-Wilk normality test of productivity distribution of occupations at steady state. All 50 replications of the 8 situations are tested, mean and standard deviation of W statistics and P-value are shown.

We define the initial condition with one functioning occupation which is self-supportable, for the demonstrating of evolving dynamics of our model society. As time goes by, other occupations are gradually generated through mutation. After sufficient steps, the society reaches its steady structure (Figure 2). This self-booting capability also means a society can repair itself if some of its occupations are destroyed for some reason. In all simulations, number of occupations and total productivity gradually increase from initial value through final steady state. Different simulations could result in different steady state productivity and transition time according to parameter values. Societies with larger *λ_pf_* and *a/b* values are more productive at their steady states, while a bigger *p_m_* value would speed up this evolving process since occupations can be generated more quickly. Owing to random effects, trajectory of different simulations with same parameter value combination may be different, which is revealed by the standard deviation of the number of occupations. The distribution of final productivity is of normal type (Figure 3-A), which we tested with the Shapiro-Walk normality test, and achieved considerable accordance (Figure 3-B).

**Figure 4:**
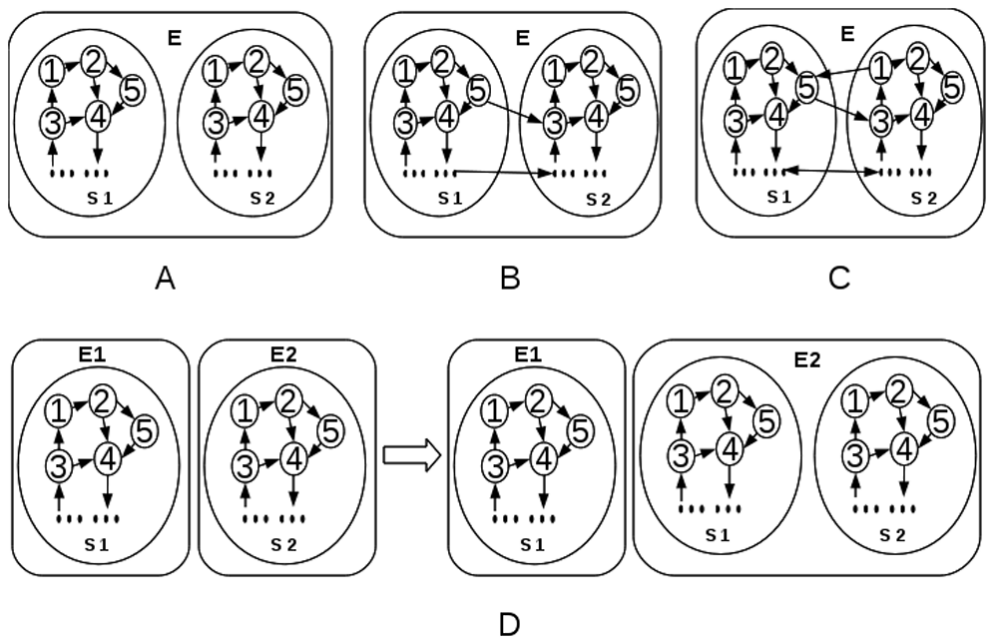
Relationships between two societies. (A) Competition between S1 and S2. (B) S1 supports S2. (C) Mutually supporting between S1 and S2. (D) S1 invades S2.

To depict societal transition patterns, some variations are made to the models to introduce in driving forces of transitions. First, society inhabitants do not only transform within the society, they could also create new occupations that do not belong to the current society. We would denote this collection as derived society (*Sd*) of the original society (*So*). However, we do not require the supporting matrix C of *Sd* to be irreducible as *So*’s. Besides, the possibility of transforming into occupations of *Sd* is lower than into *So* itself, because inhabitants are familiar with the existing occupations of their own society, but have limited knowledge of the outside world. The ability of generating new occupations is the driving force of societal evolving, which is a normal phenomenon in real world. A newly generated occupation must be able to survive (which means it must get support), or the society inhabitants won't transfer to it and thus the occupation won't be created. Usually the supporting comes from the occupations of the original society, which in turn may benefit from the new occupation or not. The structure of the derived society could be affected by the original society, the environment, and various other factors. Further exploration of this issue is out of the scope of this paper. In the following we would presume different instances without explaining how they are derived. Based on relationships between occupations, we define different types of relationships between two societies S1 and S2, which share one common environment E, as follows (Figure 4): (a) Competition. Occupations of S1 don't support occupations of S2 and *vice versa*. (b) Supporting. Some occupations in S1 support some occupations in S2, but the reverse is not true. (c) Mutually benefiting. Some occupations in S1 support some occupations in S2, and the reverse is also true. Second, environmental fluctuation would occasionally happen (like environment disasters that happen in real world), which affects all the occupations in the environment. That is, the environmental restriction will be intensified (*i.e.*, a sudden amplification of coefficient *b* in the model), and all occupations in the environment would have a difficult time. We further suppose the disturbance happens in a pulse manner, which means it only affects one step and then return back to normal state in the next time step. Third, invasion could happen when an invader society (*Si*) colonize an environment which another society already dwells in. Invasion is a kind of competition, with *Si* come from another environment. Furthermore, we suppose all the societies in our model have identical supporting coefficients (denoted by *a*) and environmental restricting coefficient (denoted by *b*) for simplicity. Besides, all the environments have identical characteristics.

## Results

We set four scenarios, *i.e.*, supporting, disturbing, invading, and mutually supporting——which could be interpreted as different kind of driving forces——to check the societal evolving dynamics. For each scenario, we set several combinations of parameter values, and each combination was run with 50 replications as in previous demonstrative running of the model. The three societal transition patterns, *i.e.*, standstill, collapse, and grow, could be revealed.

### Supporting

**Figure 5:**
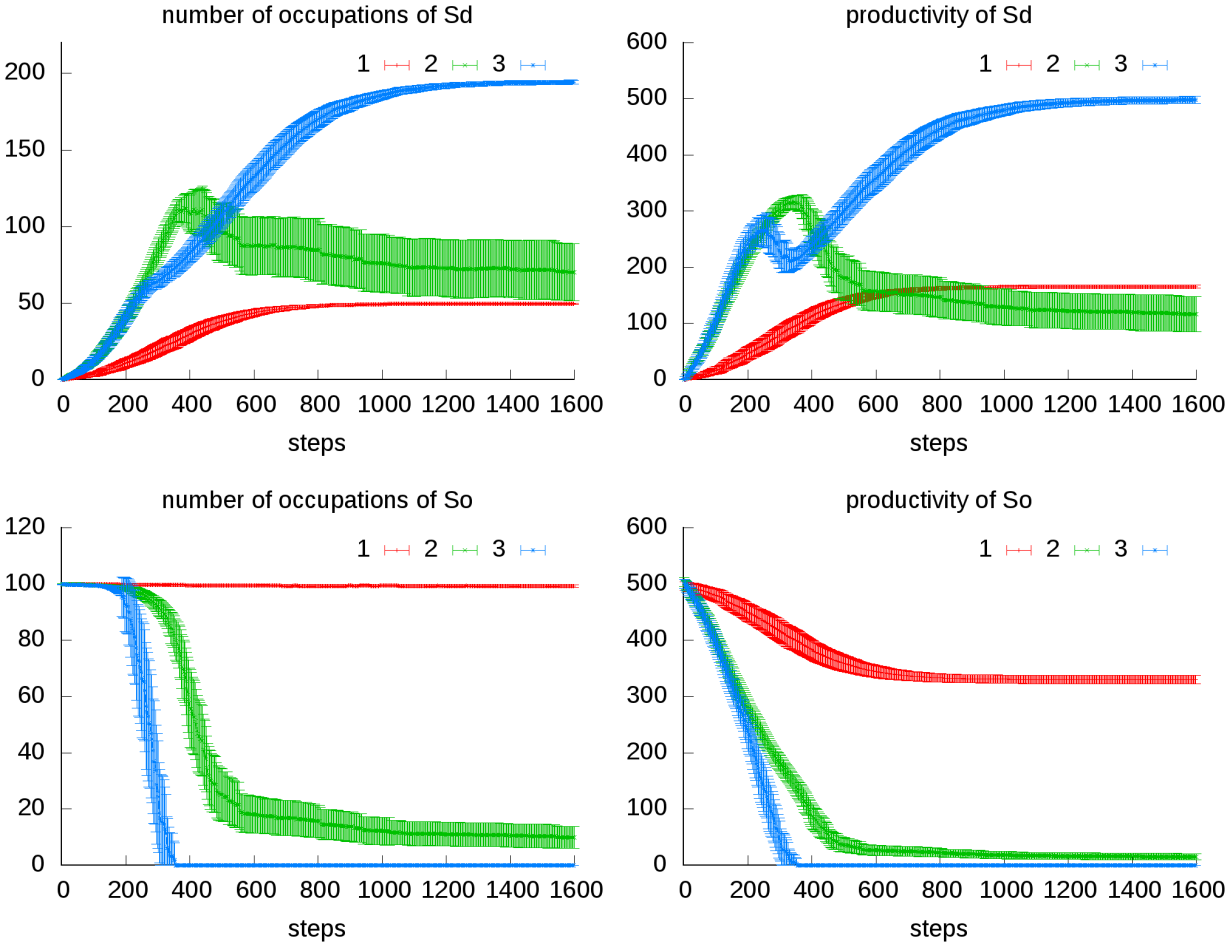
Transitional dynamics of the supporting scenario. We set three different combinations of parameter values: (1) *n_d_* = 50, *p_c_od_* = 0.1_,_ *p_c_dd_* = 0; (2) *n_d_* = 200, *p_c_od_ = *0.2_,_ *p_c_dd_* = 0; (3) *n_d_* = 200, *p_c_od_ = *0.2_,_ *p_c_dd_* = 0.05; while other parameters are kept constant (*n*_o_ = *n_d_* = 100, *p_c_oo_* = 0.1, *a/b* = 100, *p_m_* = 0.01). For each combination, 50 replications are run, and the mean and standard deviation of total productivity and number of occupations of the original society (*So*) and derived society (*Sd*) are shown. In combination 1, *So* is affordable of *Sd* that parasitize it thus result in the coexistence of *So* and *Sd*. In combination 2, *Sd* has a much larger size (*n_d_*) and the parasitism efficiency is also improved (*p_c_od_*), thus *So*’s number of occupation and productivity are greatly shrinked; in 4 of the 50 replications, *So* is completely eliminated. As a result, *Sd* also suffers a big drop of its productivity and number of occupations, or even goes extinct in the conditions that *So* is completely ruined. In combination 3, *Sd* is able to support itself, thus more competent in grabbing the productivity contributed by *So*; besides, it is able to survive after *So*’s collapse, thus result in the replacing of *So* by *Sd*. The productivity of *Sd* undergoes a short dropping before it rise again to steady value, that is because as *So* quickly collapses, its supporting to *Sd* is deprived.

The derived society *Sd* is generated and supported by the original society *So*, but does not support *So* back. That is to say, *Sd* is a parasitic society, which does not contribute to the total productivity. As *Sd* gradually forms, it would grab more and more portion of the total productivity, and thus shrinks *So*’s share of productivity. The equilibrium state depends on *Sd*’ s size, its capability of supporting itself (*p_c_dd_*) and *So*’s supporting strength to *Sd* (*p_c_od_*). The bigger these parameters, the more portion of total productivity that *Sd* would grab. If all the occupations of *So* still have their productivity above *limina* at the time *Sd* has completely formed, *So* persists. This is the situation that a society stands still. But if *Sd* grabs too much share of the limited productivity, *So* would lose parts of its occupations or even completely eliminated. If *Sd*’ s occupations are mutually supportable, it would replace *So* after its ruin; otherwise, *Sd* would follow *So*’s fate and the environment would be cleared (Figure 5).

### Disturbing

A society can suffer from environmental fluctuation, which makes the living condition of all the occupations in the society severe. Their productivity would be brought down, and some of the occupations may be eliminated if they could no longer feed the minimum practitioners required for their function. If all the occupations in the society get destroyed, the society also collapses. Suppose there are *n_0_* occupations in the society and their productivity is uniformly distributed, then the environmental restriction coefficient to wipe out the society would be:
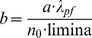
(11)


**Figure 6:**
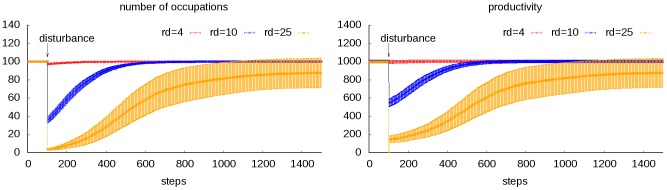
Transitional dynamics of the disturbing scenario. We set three gradients for parameter *r_d_*: 4, 10 and 25, while other parameters are kept constant (*n*_o_ = *n_d_* = 100, *p_c_oo_* = 0.1, *a/b* = 100, *p_m_* = 0.01). 50 replications are run for each combination. It could be revealed as *r_d_* increases, more productivity and occupations would be lost in the fluctuation, and more time needed for recovering. The society also suffers a greater risk of being wiped out when *r_d_* gets larger. In the condition that *r_d_*  = 25, 5 out of the 50 replications resulted in the extinction of the society, while none happened in the other two conditions.

But in current model, distribution of productivity in a society is bell shaped, which makes a difference. Some peripheral occupations with limited supporting would be eliminated easily, but it would be hard to wipe out the whole society. The failed occupations would no longer support the remaining ones, but they would not compete for resource either. After the shock, the environmental restriction coefficient returns normal, and the survived occupations would play the role of seed and rebuild the society (Figure 6).

### Invading

**Figure 7:**
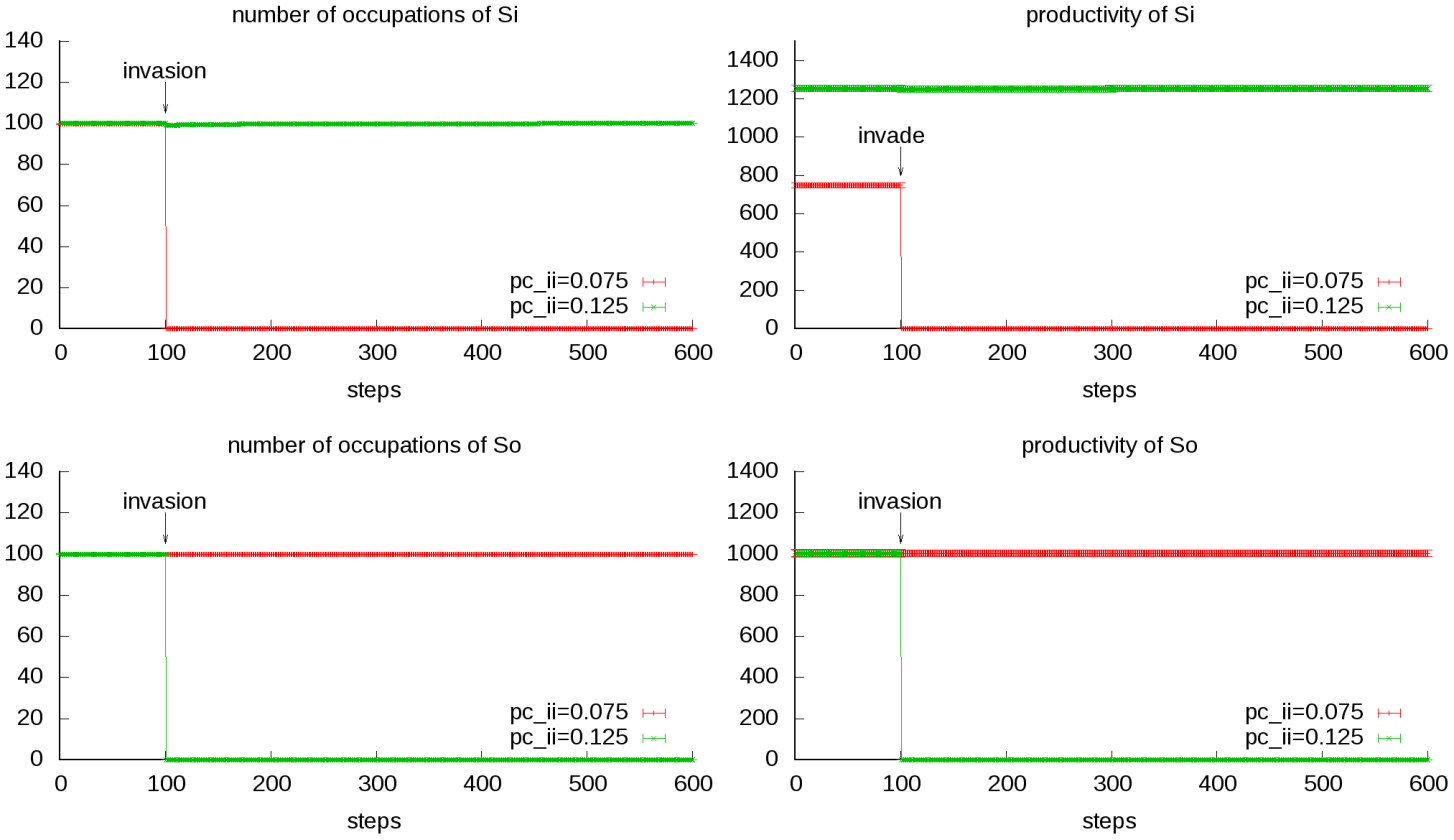
Transitional dynamics of the invading scenario. We set two gradients for parameter *p_c_ii_*: 0.075 and 0.125; while other parameters are kept constant (*n*_o_ = *n*_i_ = 100, *p_c_oo_* = 0.1, *a/b* = 100, *p_m_* = 0.01). The mean and standard deviation of total productivity and number of occupations of the original society (*So*) and invader society (*Si*) are shown. In the first situation, the invader society is not as competent as the original society, and thus the original society is not affected; while in the second situation, the invading society is more competent, causes the extinction of the original society.

In this scenario, the environment *So* originally dwells in is colonized by a squad of *Si* from another environment that would compete with *So*. The one with greater productivity will finally win and take over the environment. Here we suppose the squad that *Si* sends is intact, *i.e.*, with all occupations of *Si* in it. The invasion here is in a peaceful manner. The two societies compete with each other, but do not fight directly with each other (Figure 7).

### Mutually supporting

**Figure 8:**
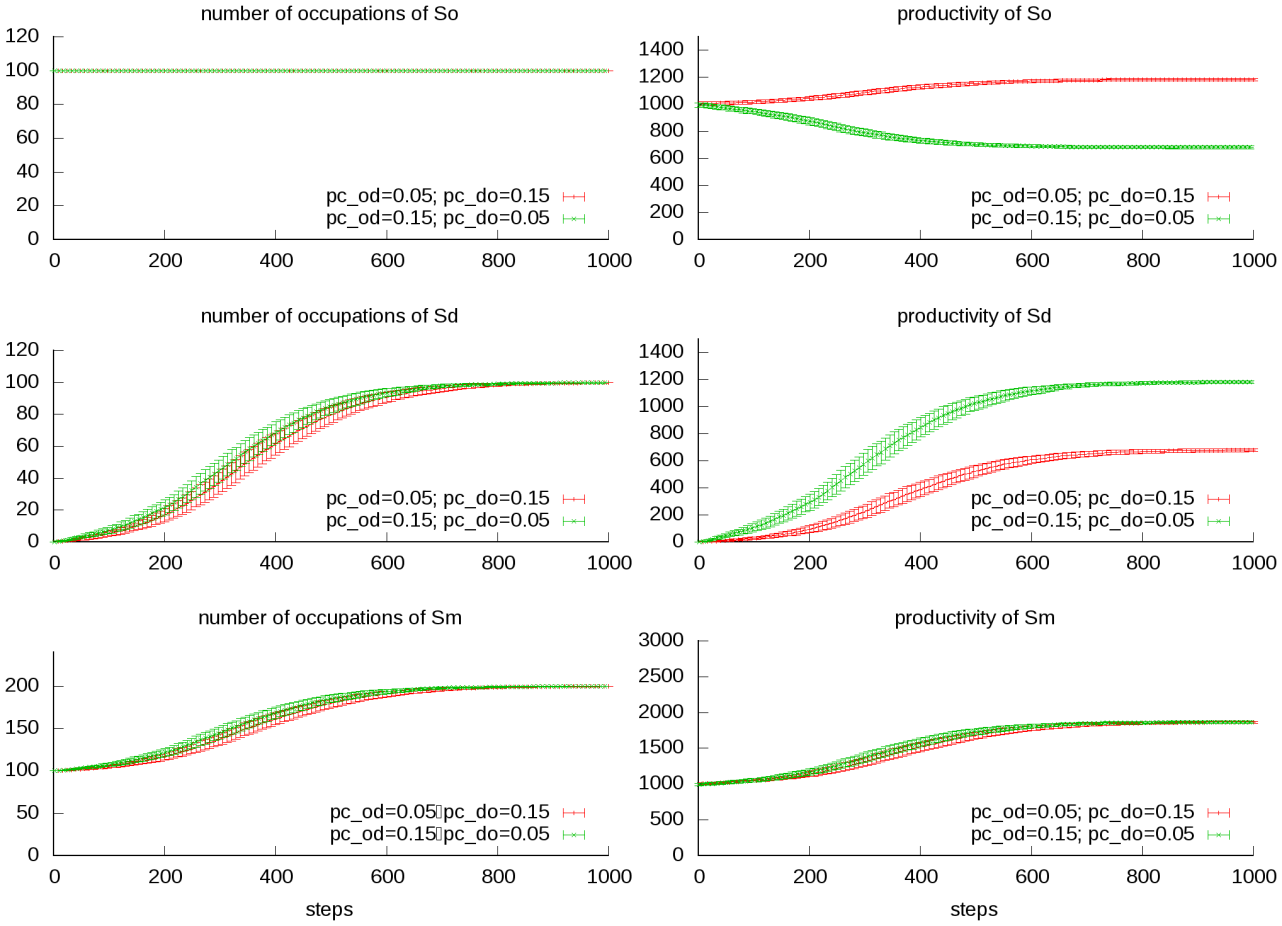
Transitional dynamics of the mutually supporting scenario. The original society and the derived society are mutually supportive, they compose a new society, which could be interpreted as growing of the original society. Situation 1: *p_c_od_* =  *p_c_do_* =  *p_c_dd_* = 0.5, situation 2: *p_c_od_* =  *p_c_do_* =  *p_c_dd_* = 1.5. Other parameters are kept constant (*n_o_* = *n_d_* = 100, *p_c_oo_* = 0.1, *a/b* = 100, *p_m_* = 0.01). The mean and standard deviation of total productivity and number of occupations of the original (*So*), derived (*Sd*) and merged society(*Sm*) are shown. Productivity of the occupations of the original society could decrease (situation 1) or increase (situation 2) during the growing process due to different supporting strength.

If the generated society supports the original society back, society growth would be achieved. By our previous definition, the original society and the derived can merge into a new one, and the supporting relationships of this new society form an irreducible matrix. Growth can happen gradually or abruptly. When technology breakthrough happens, new occupations can be created in large quantities (Figure 8).

## Discussion

Real societies are more complex. It is much easier to get to the details than come up with a comprehensive concept model, which is more valuable when we are concerning about the future sustainability of our civilization. In current model, we define society as occupations interacted within a specific environment, which is characterized by structure and productivity. Evolving dynamics under different driving forces have been simulated. The results show that the model exhibits the three transition patterns with considerable accordance. Standstill could be reached under certain conditions of supporting scenario; growth could be realized under mutually supporting scenario; and many possibilities exist for the collapse of a society.

One key fact about complex real world systems is that they always change; there is no such thing as “permanent structure”. As to a society, one common change is the inevitable adhesion of new occupations to it, which could be somewhat attributed to relentless dissatisfaction and creativity of mankind. This we regard as a main driving force of societal transition, while our model result shows that the effect is determined by the characters of the new occupations. Parasite occupations “supported by the original society but do not support back” would harm the society in long term when they are abundant (except those occasions when some further occupations mediated the back loop supporting). If the scale of parasite occupations is limited, the original society could persist; but if their scale is very big and sprawling, the original society would be eliminated. On the other side, for those societies with newly created occupations mutually supportive with the original society, the productivity increases and the society would expand and become more robust. This could be compared to the process of industrialization. The industrial part of the society grows out of the original agricultural society, once it gets mature, the productivity of agriculture can be greatly promoted because of the support from the industry departments.

The average productivity of each occupation is an important indicator. Normally, occupations would have their productivity higher than *limina*, and the surplus portion could be regarded as average reserve of this society. When compared to reality, primitive societies usually have simple structure and limited “reserve”, which probably leads to their high risk. For example, inhabitants of societies that live on gathering and hunting are more likely to suffer starvation than those that have developed agriculture. However, although advanced societies could have abundant reserve, it could be gradually dissipated by parasites, which would shrink the share of productivity of each occupation close to *limina* and thus bring the society into risk. The more reserve a society originally has, the more robust it would be in parasitism enduring. The average reserve in the current model could be given by Equation 12.
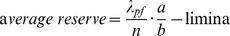
(12)


Many dynastic cycle models imbed carrying capacity in them (*e.g.*, land constraint and diminishing marginal productivity [19]). As the society develops and approaches environmental limit, adverse effects begin to accumulate, usually leading to vicious cycles and finally collapse of the society. However, the concept of carrying capacity does not always apply, since real societies might successfully escape from this trap by technology development and concomitant structure transformation. Thus, we believe carrying capacity is better considered as a variable, which changes along with the structure of the society. Usually, a society has seeds of both diminishing and increasing return imbedded in it, and it is the tradeoff between these two effects that dominates the course of the society. Many economists noticed the prominence of these two effects. For example, Adam Smith addressed that specialization and division of work is the source of economy growth, while the wealth of a nation would reach its maximum when the potentials of its land and climate have been used up [32]. Schumpeter [33] combined the two effects and proposed the “creative destruction” theory, illustrating the business cycles of Capitalism society. In our opinion, diminishing return is a normal and predictable trend, while increasing return is hardly sustainable and could not be expected in long term. That is, each increasing return process would finally be restricted by diminishing return, and there is no known mechanism that leads to sustainable increasing of return [34], [35]. In the present model, these two effects are modeled by introducing in different types of new occupations to the society and two arrangements in this process. First, the parameter *p_c_xy,_* which is the probability of an occupation in society *x* supporting an occupation in society *y* (here we suppose *x* to be the original society), is considerably small. Second, the environmental restriction is an increasing function of total population, meaning the marginal return the society receives is digressive by adding new occupations mutually supportive with original occupations. The spirit of this mechanism is embodied in the aforementioned complex system theories like SOC [8], DPE [10], Adaptive Cycle [12], and Tainter’s societies evolving theory [4], while we explicitly demonstrated it. Thus, our model also serves as an illustration for these theories.

Environmental fluctuation is another important driving force of societal transition. Extreme examples are Pompeii and Herculaneum, which were buried in eruption of Mount Vesuvius in AD 79 [36]. Alternatively, recent study found that modest rainfall reduction might be the tipping point to disintegration of the Classic Maya civilization in the Yucatan Peninsula and Central America [37]. In many occasions, disturbance would cause damages, but not collapse a society. In current model, disturbance is introduced by a sudden increase in environmental restriction coefficient, thus is indirect and transient. Simulation results show that disturbance severity is critical, while distribution of productivity of occupations is also of great significance, for it represents relative robustness of the occupations. This distribution is determined by the structure of the society, which is randomly generated in the model.

Invasion is also a widely known driving force of societal collapse. The definition of invasion in the model is actually peaceful colonization, with no direct conflict between two societies. Besides, the invader society and the target society should have same environmental characteristics, and no supporting happens between any occupations of the two societies. These assumptions are a bit rigorous, and could be relaxed in the model. The relaxation of the first condition results in different environmental restriction coefficients that would affect competition; relaxation of the second condition results in combined effects of competition and mergence which leads to some kind of new society.

Combining different driving forces is possible, which can be easily implemented in the current model. For example, disturbance could be combined with invasion. A society suffering disturbance may provide opportunity for invaders because its competitive power is lessened. Another common combination is parasitism and disturbance. As parasitic occupations accumulate in a society, the living condition would be lowered and thus more sensitive to disturbance. This could be compared to the situation in ancient China. Natural disaster of same severity would cause greater damage to a dynasty during the second half of its lifecycle than the first half [38]. Moreover, it is also possible to combine generation of mutually supportive occupations with loosening of environmental restriction, which may result in leap forward growth of a society. This could be compared to the boom of capitalist society in Europe during the 17^th^ and 18^th^ century. Decentralized structure of the society provided space for development of capitalist occupations, while discovery of the “New World” provided it with resources, loosening the environmental restriction [39].

Our study also provides insights into understanding sustainable development. The whole human civilization is composed of interactive societies; the evolving dynamics could be compared to biological evolution. Similar to natural selection, history selects societies that can survive various impacts and confrontations, so that our civilization as a whole gets more complex and productive. Underneath enormous delicate species flourishing today, there is even incredibly large number of extinct ones. Likewise, successfully sustained societies account for only a tiny fraction compared with fading ones. However, owing to the great advance of communication and transportation technology, previously isolated societies are now merging quickly and global society is in forming which would be the only existing society in the world. Thus selection is abolished. If this society goes extinct, the whole civilization ends. Here rise the question which we believe is critical to sustainable development: how do we avoid the fate of collapse by gradually accumulating those parasitic components and other types of structure change? It should be notified that although some societies have persisted for a very long time, it does not guarantee their survival forever. Jacob [40] compared evolution to the work of a tinkerer, emphasizing that it is a contingent and unpredictable process. This also applies to the evolution of human society, which means we have to expect the modern society work like a master craftsman if we are to achieve sustainability. Levin [41], [42] points out that Gaia hypothesis, which depicts the biosphere as a self-regulating system of organisms and their environment [43], is defective. The biosphere is but a complex adaptive system. The efficacy of selection is rather limited at the level of whole society and biosphere, and the system may finally stop to function like an old broken truck. Modern society has many times run into big crises, which might be largely attributed to accumulated structure change and environmental limit that lead to low per capita productivity, and it was technological revolution that prevents us from falling into downward spiral. However, real society is way complex and dynamic, mechanisms may exist that can help sustain our civilization.

Improvements could be made to the present model which would endow it with the power of simulating more society evolving dynamic patterns. For example, we can assign different values to the supporting coefficients and make them subject to occupation mutations so as to simulate consecutive productivity enhancement and structure change through substitution of old occupations with new ones. Furthermore, overshoot could also be introduced into the model by connecting productivity with environmental fluctuation frequency and severity, *i.e.*, as productivity gets higher, the frequency and severity of fluctuation would also rise. In addition, there are also characteristics not covered in our model that could be important in real society evolving. For example, spatiality, negative interactions (*e.g.* crimes), and multi-dimensionality of environment characteristics would be necessary to depict societies more accurately and to address some societal transition behaviors properly (*e.g.*, migration). Besides, other modeling approaches would also be beneficial. More endeavor in this area is favored.

## Materials and Methods

Our model is implemented with the c programming language, compiled with Gcc 4.4.5, under the debian 6 linux platform with an ordinary personal computer. Charts in this paper are plotted with Gnuplot 4.4. Source codes of the model with necessary descriptions are available as supporting information to this paper (Source code S1).

## Supporting Information

Source code S1 **The source codes for the model.** This is a collection of 5 folders, each contains a collection of source files needed for compiling and running the preliminary demonstration or one of the four scenarios described in the model. (1) preliminaryDemo: elementary demonstration of the model; (2) support: the supporting scenario; (3) disturb: the disturbing scenario; (4) invade: the invading scenario; (5) mutualsupport: the mutually supporting scenario.
(ZIP)
